# Preoperative versus postoperative ultrasound-guided rectus sheath block for improving pain, sleep quality and cytokine levels of patients with open midline incisions undergoing transabdominal gynaecological operation: study protocol for a randomised controlled trial

**DOI:** 10.1186/s13063-015-1096-0

**Published:** 2015-12-10

**Authors:** Feng Jin, Xiao-qian Li, Wen-fei Tan, Hong Ma, Huang-wei Lu

**Affiliations:** Department of Anaesthesiology, the First Hospital of China Medical University, Shenyang, China

**Keywords:** Pain, Rectus sheath block, Sleep, Cytokines

## Abstract

**Background:**

Rectus sheath block (RSB) is used for postoperative pain relief in patients undergoing abdominal surgery with midline incision. Preoperative RSB has been shown to be effective, but it has not been compared with postoperative RSB. The aim of the present study is to evaluate postoperative pain, sleep quality and changes in the cytokine levels of patients undergoing gynaecological surgery with RSB performed preoperatively versus postoperatively.

**Methods/Design:**

This study is a prospective, randomised, controlled (randomised, parallel group, concealed allocation), single-blinded trial. All patients undergoing transabdominal gynaecological surgery will be randomised 1:1 to the treatment intervention with general anaesthesia as an adjunct to preoperative or postoperative RSB. The objective of the trial is to evaluate postoperative pain, sleep quality and changes in the cytokine levels of patients undergoing gynaecological surgery with RSB performed preoperatively (*n* = 32) versus postoperatively (*n* = 32). All of the patients, irrespective of group allocation, will receive patient-controlled intravenous analgesia (PCIA) with oxycodone.

The primary objective is to compare the interval between leaving the post-anaesthesia care unit and receiving the first PCIA bolus injection on the first postoperative night between patients who receive preoperative versus postoperative RSB. The secondary objectives will be to compare (1) cumulative oxycodone consumption at 24 hours after surgery; (2) postoperative sleep quality, as measured using a BIS-Vista monitor during the first night after surgery; and (3) cytokine levels (interleukin-1, interleukin-6, tumour necrosis factor-α and interferon-γ) during surgery and at 24 and 48 hours postoperatively.

**Discussion:**

Clinical experience has suggested that RSB is a very effective postoperative analgesic technique, and we will answer the following questions with this trial. Do preoperative block and postoperative block have the same duration of analgesic effects? Can postoperative block extend the analgesic time? The results of this study could have actual clinical applications that could help to reduce postoperative pain and shorten hospital stays.

**Trial registration:**

Current Controlled Trials NCT02477098 15 June 2015.

## Background

Multimodal analgesia techniques can be used to provide adequate analgesia for abdominal surgery with midline incision, with the goal of limiting postoperative pain. Each new technique must meet increasing demands for safety and effectiveness. Pain after abdominal surgery has two components, somatic and visceral, both of which are initiated by nociceptor stimulation [1].

Reliance on high-dose opiates to obtain adequate postoperative analgesia has been strongly associated with postoperative nausea and vomiting, sedation and ileus [2, 3], all of which can delay postoperative recovery. Epidural analgesia (EA) has been reported to result in complications such as nerve injury, epidural haematoma and low blood pressure [4]. Additionally, some patients are unsuitable for EA, such as those with coagulopathy and sepsis, those with limited movement and those who are uncooperative [5]. Further, the high reported failure rate cannot be ignored [6]. Transversus abdominis plane (TAP) block has become popular in recent years. However, there is no guarantee of satisfactory block for midline incision [7, 8].

Rectus sheath block (RSB) was first introduced into clinical practice in 1899; as described by Schleich, it is a regional anaesthetic technique that involves blocking of the ventral rami of the 7th to 12th intercostal nerves by injection of local anaesthetic into the space between the rectus muscle and posterior rectus sheath to achieve analgesic effects [9, 10]. With the development of long-acting local anaesthetic agents and ultrasonic technology, RSB has become safer and more effective [11, 12]. Recently, it has been widely used for postoperative analgesia, including that after laparoscopy, umbilical hernia repair, paediatric surgery and gynaecological surgery [13–17]. However, better analgesic effects can be achieved when incisions are limited to the midline, and RSB only provides analgesia for somatic pain but does nothing to alleviate pain of visceral origin [18].

An early finding that less oxycodone than morphine is needed for visceral pain relief after major abdominal surgery [19] has been confirmed in patients undergoing laparoscopic gynaecological surgery [20]. Oxycodone is a semisynthetic thebaine derivative μ-opioid receptor agonist that has been in clinical use since 1917, and it has been the most commonly used analgesic in Finland for the management of postoperative and other acute pain in adults since the 1960s [21]. Over the past two decades, the use of oxycodone has surpassed that of morphine in several countries [22].

In our recent clinical observation, the combination of RSB and patient-controlled intravenous analgesia (PCIA) with oxycodone has been shown to be a potentially better multimodal analgesic technique for patients undergoing transabdominal gynaecological surgery with open midline incision. Thus, this combination treatment might alleviate both somatic and visceral pain. However, it is unknown how this technique influences postoperative sleep quality and cytokine levels.

During the first postoperative nights, sleep patterns can be severely disturbed, with marked decreases in total sleep time, slow-wave sleep duration, and rapid eye movement sleep, which are features associated with frequent sleep arousals [23–29]. The pronounced reduction in sleep during the first nights following major surgery can affect recovery [30]. Sleep disturbance after surgery can be caused by factors such as the anaesthesia used, the environment after surgery, pain and sympathetic activation, and psychological responses [24]. Sound sleep impacts adaptive immune functions. Specifically, the acute effects of regular sleep have been shown to have lasting influences on cytokine levels compared with 24 hours of continuous wakefulness [31–33]. Surgical stress accompanied by general anaesthesia is believed to suppress immunity, and surgery can influence the levels of secreted cytokines, which are responsible for suppressing cell-mediated immunity after surgery [34]. In addition, anaesthetics and sedative agents themselves are known to have immunomodulatory activities [35]. However, little information is available about the use of regional block as an RSB adjunct in addition to the effects of oxycodone on sleep quality and immune function during the immediate postoperative period.

RSB is usually performed before surgery because it inhibits reflection of the skin incision and reduces the amount of intraoperative anaesthetic used [36–38]. While postoperative surgical RSB appears to provide effective postoperative analgesia for patients undergoing major gynaecological surgery [39], there have been no randomised, controlled trials comparing the differences in immune function of patients receiving RSB before versus after surgery.

We hypothesised that performing RSB after surgery might result in a longer duration of analgesic effects and have a subtle influence on sleep quality after surgery but that it will not decrease the perioperative cytokine levels of patients undergoing gynaecological surgery. The present report will follow the guidelines of the Standard Protocol Items: Recommendations for Interventional Trials (SPIRIT).

## Methods/Design

### Study design and outcomes

This study is a prospective, randomised, controlled (randomised, parallel group, concealed allocation), single-blinded trial. All patients undergoing transabdominal gynaecological surgery will be randomised 1:1 to the treatment intervention with RSB preoperatively or postoperatively. The objective of the trial is to evaluate the postoperative pain, sleep quality and changes in cytokine levels of patients undergoing gynaecological surgery with RSB performed preoperatively versus postoperatively.

The primary objective is to compare the interval between leaving the post-anaesthesia care unit (PACU) and receiving the first PCIA bolus injection on the first postoperative night between patients who receive preoperative RSB and those who receive postoperative RSB. The secondary objectives will be to compare the following: (1) cumulative oxycodone consumption at 24 hours after surgery between patients who receive preoperative versus postoperative RSB; (2) postoperative sleep quality, which will be measured using a bispectral index (BIS)-Vista monitor during the first night after surgery [40, 41]; and (3) cytokine levels (interleukin-6 (IL-6), tumour necrosis factor-α (TNF-α), IL-1 and interferon-γ (IFN-γ)), which will be compared during the operation and at 24 and 48 hours postoperatively.

### Study setting and population

#### Population

Patients scheduled for elective transabdominal gynaecological surgery at the First Hospital of China Medical University will be recruited for the study beginning in September 2015. The purpose, procedures, and potential risks and benefits of the study will be explained to the participants, and written informed consent will be obtained.

#### Inclusion and exclusion criteria

The inclusion criteria are as follows: (1) an age of 18 to 65 years old; (2) scheduled to undergo elective midline incision transabdominal gynaecological surgery for a benign mass; and (3) American Society of Anaesthesiologists (ASA) risk classification I–II.

**Fig. 1:**
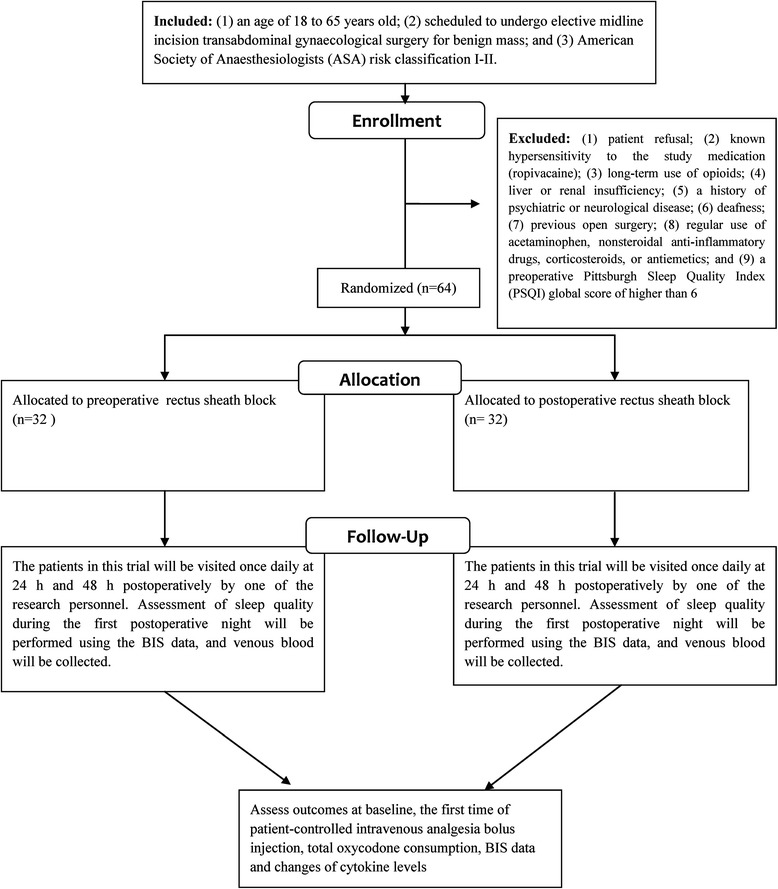
Flow chart of the study

The exclusion criteria are as follows: (1) patient refusal; (2) known hypersensitivity to the study medication (ropivacaine); (3) long-term use of opioids; (4) liver or renal insufficiency; (5) a history of psychiatric or neurological disease; (6) deafness; (7) previous open surgery; (8) regular use of acetaminophen, nonsteroidal anti-inflammatory drugs, corticosteroids, or antiemetics; and (9) a preoperative Pittsburgh Sleep Quality Index (PSQI) global score of higher than 6 (Fig. 1).

#### Ethical aspects and informed consent

The trial was approved by the Ethics Committee of the First Hospital of China Medical University (protocol number 2014071701, Chairman Professor Jian Kang) and was registered with the Clinical Trials Registry (NCT02477098). All of the participants will provide written informed consent in accordance with the Declaration of Helsinki.

#### Single-blind randomization and allocation concealment

All patients requiring transabdominal gynaecological surgery will be randomly assigned via computer-generated sequences placed into sealed envelopes to the following two groups: a general anaesthesia group with preoperative RSB (Group PRE); and a general anaesthesia group with postoperative RSB (Group POST). Treatment allocation will be revealed by opening the envelope on the morning of surgery. All of the patients, in addition to the staff involved in postoperative data collection and analyses, will be blinded to the group allocations. The trial will be monitored by an independent data and safety monitoring organization. The group allocations will not be revealed until the final statistical analysis is completed.

### Interventions

#### Before anaesthesia

All patients will be assessed with the PSQI 1 day before the operation [42]. The PSQI differentiates between good sleepers (PSQI global score < 6) and poor sleepers (PSQI global score ≥ 6) with high sensitivity and specificity. The patients will receive routine general anaesthesia and surgery in this study. No additional requirements or preoperative oral analgesics will be permitted. When the patients are in the operating room, standard monitoring will be performed, including evaluations of systolic blood pressure, diastolic blood pressure, heart rate, electrocardiography, blood oxygen saturation and the BIS. After venipuncture, 3 ml of venous blood will be collected for plasma inflammatory cytokine measurements.

#### General anaesthesia

General anaesthesia will be induced as follows: 2 mg/kg propofol given intravenously (IV); 2 mg midazolam given IV; 0.4 μg/kg sufentanil given IV; and 0.2 mg/kg cisatracurium given IV. The patient’s lungs will be ventilated with intermittent positive pressure. The tidal volume will be adjusted to 6–8 ml/kg, and the ventilator rate will be adjusted to maintain end-tidal CO_2_ at 35–45 mmHg. For maintenance of anaesthesia, sevoflurane (Baxter Healthcare of Puerto Rico, Guayama, Puerto Rico) will be used at an end-tidal concentration of 2–2.5 %, and an air-oxygen (FiO_2_: 50 %) mixture will be adjusted for the maintenance of anaesthesia with intraoperative titration of sufentanil given IV, according to a BIS value of 40 to 60, after intubation in both groups. A 0.05 mg/kg dose of additional cisatracurium will be applied if needed. At the end of surgery, the trachea will be extubated after the return of spontaneous respiration and neuromuscular function, and the patient will then be transferred to the PACU. The patients will receive pain relief (visual analogue scale, VAS, score of less than 3) using 5–10 μg titrated sufentanil. Three millilitres of venous blood will be collected for plasma inflammatory cytokine measurements at the PACU.

#### RSB technique

Before surgery (Group PRE) or at the end of surgery (Group POST), a bilateral single-shot RSB will be performed under ultrasound guidance by one anaesthesiologist. The rectus muscle will be imaged with an ultrasound probe in the transverse orientation at the level of the umbilicus. A broadband (5–12 MHz) linear array ultrasound probe (S-Nerve Ultrasound System, Sonosite, Bothell, Washington, USA) will be used with an imaging depth of 4–6 cm. A PAJUNK (PAJUNK, GmbH, Medizintechnologie, Geisingen, Germany) insulated needle (50-mm, 21-gauge) will be introduced a few millimetres from the probe using an in-plane technique at an angle of approximately 45° to the skin. The ultrasound imaging will allow for identification of the rectus muscle and two hyperechoic railway-like lines deep in this muscle (the posterior rectus sheath and peritoneum). Under direct vision, the needle tip will be advanced to the desired position, where 20 ml of 0.5 % ropivacaine hydrochloride will be injected, causing hydrodissection of the rectus muscle away from the posterior rectus sheath. The technique will be repeated on the opposite side. It will be performed by one investigator using a completely aseptic technique.

#### Postoperative analgesia: PCIA

All of the patients, irrespective of the group allocation, will receive PCIA with oxycodone. A PCIA pump (AponZZB-I50, Nantong, China) will be set up with a bolus injection of 2 mg every 5 minutes for a maximum of 10 mg every 4 hours without basal infusion. The PCIA pump will be stopped on the third postoperative day, and postoperative nausea and vomiting will be treated with 5 mg tropisetron given IV (on the ward).

#### Follow-up visits

The patients in this trial will be visited once daily at 24 and 48 hours postoperatively by one of the research personnel. Assessment of sleep quality during the first postoperative night will be performed using the BIS data, and venous blood will be collected.

#### End of participation in the study

Patients will be excluded from the study for the following reasons: (1) refusal to participate; (2) an exclusion criterion identified during the course of the intervention; and/or (3) the occurrence of a severe adverse event.

#### Criteria for removal from the study

During the study, patients found to have the following criteria will be removed from the study: (1) the loss of over 500 ml of blood during surgery; (2) an operation time of longer than 3 hours; (3) a violation of the trial protocol; (4) an unacceptable risk of a serious adverse event; or (5) desire to withdraw from the study.

#### Assessment of safety

The interventional treatment will be administered to patients under standard monitoring in a fully equipped operation room. It is possible that immediate detection of adverse events will occur, in which case they will be treated immediately. However, administration of RSB will be immediately stopped in cases in which the study participant shows significant deterioration. Moreover, the inclusion of each individual patient in the study will be indicated in the electronic hospital information system and hence will be visible to all the physicians and nurses involved in the care of the patient. This visibility will facilitate the reporting of adverse events to the principal investigator. The principal investigator will report suspected unexpected serious adverse reactions to the health authorities.

#### Data collection and management

Trained staff will record all of the data and complete a trial-specific electronic case report form (eCRF). At the time of patient inclusion, patient data on demographic characteristics and history of past illnesses will be collected, including age, height and weight, and diagnosis. Intraoperative data on the patients, including the operation start time, durations of anaesthesia and surgery, blood pressure, heart rate, volume loading, amount of sufentanil consumed, and plasma concentrations of IL-1, IL-6, TNF-α, and IFN-γ, will be collected at the following time points: at baseline (before induction), upon arrival at the PACU, and at 24 and 48 hours postoperatively.

The time of the first PCIA bolus injection and the amount of cumulative PCIA consumption over 24 hours postoperatively will be recorded.

**Fig. 2:**
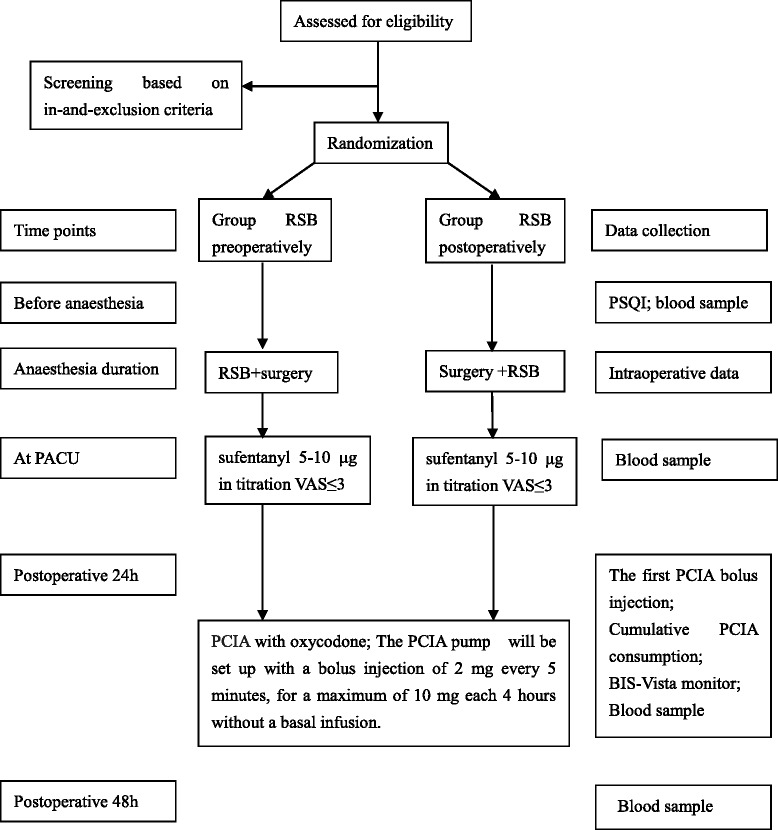
The trial procedures flow sheet. *BIS* bispectral index, *PACU* post-anaesthesia care unit, *PCIA* patient-controlled intravenous analgesia, *PSQI* Pittsburgh Sleep Quality Index, *RSB* rectus sheath block, *VAS* Visual Analogue Scale

Furthermore, sleep-related baseline data, including the PSQI score for the previous month, will be recorded. Postoperative sleep quality will be measured using the BIS data for the first night after surgery (Fig. 2).

#### Sample size

Sample size was calculated on the basis of the average (mean ± standard deviation (SD)) of the interval between leaving the PACU and their receipt of the first PCIA bolus injection on the first postoperative night calculated in the pilot study (Group PRE: 203.2 ± 21.5 minutes; and Group POST: 258.2 ± 98.7 minutes). The formula for determining sample size [43] was *n* = 15.7/*ES*^2^ + 1, where *ES* is the effect size, defined as the difference between the groups divided by the mean of the SD between the groups, with *α* = 0.05 and power = 0.8. The study is adequately powered, with *n* = 32 for each group.

#### Statistical analysis

Statistical analysis will be performed using SPSS software, version 18 for Windows (SPSS, Inc., Chicago, IL, USA). A fully specified statistical analysis protocol will be written in an independent manner. Continuous data will be described as the mean (SD or median (25 and 75 % percentiles) and will be analysed with the independent *t* test or the Mann-Whitney *U* test, respectively. Categorical data will be described as a frequency or percentage and will be analysed by the chi-square test. Repeated analysis of variance (ANOVA) will be used to determine cytokine levels within and between the groups. Before statistical testing, each continuous variable will be analysed to determine whether it has a normal distribution using the Kolmogorov-Smirnov test. Nonparametric data will be assessed using Kruskal-Wallis ANOVA or the Mann-Whitney *U* test as appropriate.

#### Dissemination policy

According to the standard protocol guidelines, the authors declare that unblinded data of the trial will not be presented prior to the release of the main results. Unblinding will occur at the end of the study. A clinical article will be written on the primary and secondary outcomes of the study, and the results will be disseminated regardless of the magnitude or direction of effect. A full study report, an anonymised participant-level dataset and the statistical code for generating the results will be made publicly available no later than 3 years after termination of the study. The present trial is not industry initiated.

## Discussion

Clinical experience has suggested that RSB is a very effective postoperative analgesic technique, and we will answer the following questions with this trial.

Do preoperative block and postoperative block have the same duration of analgesic effects? Can postoperative block extend the analgesic time? In theory, a single dose of local anaesthetic usually has a maximum duration of 12 hours [9], and if we delay the timing of the block, it should extend the duration of action after surgery. However, we must consider the original anatomical structures that are changed by surgery and whether these changes will alter the effects of block. To address this problem, we will compare the analgesic effects of PCIA, which will provide specific analgesia for both visceral and somatic pain after surgery in both groups. The indicators that we will record will include the time to administration of first rescue opiate and the number of self-controls.

The results of this study could have actual clinical applications that could help to reduce postoperative pain and shorten hospital stays. In addition, the increasing number of operations performed requires the rapid and accurate delivery of regional block anaesthesia. If good results can be obtained with postoperative block, then we can perform it at the PACU after surgery to speed up the operation flow. To minimise the imbalance of anaesthesiologists and gynaecologists, the same group of gynaecologists will perform the standard surgical procedures, and all anaesthesia will be administered by one anaesthesiologist. The limitation of our study is that we will evaluate only a single-dose RSB because a continuous infusion catheter cannot be used due to the current technical level and higher cost. However, it could be attempted in future studies.

## Trial status

Patient recruitment will start in September 2015. The predicted study completion date is December 2016.

## Abbreviations

ANOVA: analysis of variance
ASA: American Society of Anaesthesiologists
BIS: bispectral index
EA: epidural analgesia
eCRF: electronic case report form
IFN: interferon
IL: interleukin
IV: intravenous
PACU: post-anaesthesia care unit
PCIA: patient-controlled intravenous analgesia
PSQI: Pittsburgh Sleep Quality Index
RSB: rectus sheath block
SD: standard deviation
SPIRIT: Standard Protocol Items: Recommendations for Interventional Trials
TNF: tumour necrosis factor
VAS: Visual Analogue Scale
